# Clinical profile and characteristics of eosinophilic esophagitis patients presenting with refractory gastroesophageal reflux disease in Makassar, Indonesia

**DOI:** 10.11604/pamj.2022.41.93.31341

**Published:** 2022-02-02

**Authors:** Mariska Regina Kaurrany, Muhammad Amsyar Akil, Abdul Qadar Punagi, Andi Muhammad Lutfi Parewangi

**Affiliations:** 1 Department of Otorhinolarynology, Head and Neck Surgery, Faculty of Medicine, Universitas Hasanuddin, Makassar, South Sulawesi, Indonesia,; 2 Department of Internal Medicine, Faculty of Medicine, Universitas Hasanuddin, Makassar, South Sulawesi, Indonesia

**Keywords:** Eosinophilic esophagitis, peripheral blood eosinophil, refractory GERD

## Abstract

Refractory Gastroesophageal Reflux Disease (GERD) is a typical GERD that does not respond to Proton Pump Inhibitor (PPI) treatment for 8-12 weeks. One of the differential diagnoses for refractory GERD is eosinophilic esophagitis which is characterized by eosinophilic infiltration into the esophagus endothelium. However, to date, eosinophilic esophagitis is still poorly understood and data is still limited. The aim was to describe the profile and characteristics of patients with eosinophilic esophagitis presenting with refractory GERD in Makassar, Indonesia. This descriptive study involved patients with refractory GERD from two hospitals. In addition to basic demographic data, history, and body mass index, all subjects underwent peripheral blood tests to measure eosinophil level and flexible esophagoscopy, from which esophageal biopsy was done to assess the tissue eosinophil level. Eosinophilic esophagitis was established based on the examination of >15 eosinophils/high power field. Out of 32 subjects, two subjects were diagnosed with eosinophilic esophagitis (6.3%). Both subjects were male with normal BMI in the age range of 36-55 years and none had peripheral blood eosinophilia. Symptoms of eosinophilic esophagitis were similar to refractory GERD. Peripheral blood eosinophilia was not associated with incidence of eosinophilic esophagitis.

## Introduction

Gastroesophageal reflux disease (GERD) is defined as repeated reflux of gastric contents into the esophagus, resulting in disturbing symptoms and/or complications which may lead to disturbance to the patient's quality of life [1]. Symptoms experienced by patients can vary, such as non-cardiac chest pain, bloating, nausea, painful swallowing, early satiety, and heartburn, with or without typical reflux symptoms [2]. Refractory GERD is a typical GERD that does not respond to twice daily administration of proton pump inhibitor (PPI) for 4-8 weeks [3]. Identifying patients with refractory GERD is essential as upper GI endoscopy is indicated to exclude peptic ulcer disease or cancer and identify the presence of esophagitis [4]. On endoscopic examination of the upper gastrointestinal tract, two subsets of patients can be identified: patients with erosive esophagitis and patients with disturbing reflux symptoms. Patients with erosive esophagitis are characterized by esophageal mucosa damage on endoscopic examination (Erosive Reflux Disease/ERD) while patients with disturbing reflux symptoms do not show any signs of esophageal mucosa damage (Non-Erosive Reflux Disease (NERD). Based on pH monitoring, response to acid suppression, and a positive Bernstein test, available data suggests that symptoms experienced by NERD patients are also acid-induced [5]. Some causes of refractory GERD include GI hypersensitivity, eosinophilic esophagitis, and Zollinger Ellison syndrome [6].

Eosinophilic esophagitis is defined as eosinophilic infiltration into the esophagus endothelium [7]. The diagnosis can be made based on the clinical symptoms and the presence of >15 eosinophils per high power field (HPF) in the esophageal endothelium from esophageal biopsy, especially in the mid or proximal esophagus [8]. Other examinations such as barium examination and endoscopic examination can be carried out to aid diagnosis [8]. To date, due to the lack of diagnostic criteria, data on eosinophilic esophagitis is still scarce. Information about this disease is better delineated in children than adults. The exact prevalence is unknown, but several studies have found an estimated prevalence of about 2.5/100,000 adults in the United States and children about 4.3/10,000,000. This disorder often occurs in the Caucasian race, especially in male [9]. However, unfortunately, in Indonesia, study on eosinophilic esophagitis is yet to be available. Owing to the current limitation in the understanding of eosinophilic esophagitis, this study aims to report the profile and characteristics of patients with eosinophilic esophagitis presenting with refractory GERD in Makassar, Indonesia.

## Methods

**Study design and setting:** this descriptive study was done on participants presenting to the Gastroenterohepatology outpatient clinic of Wahidin Sudirohusodo Hospital and Ibnu Sina Hospital, Makassar, South Sulawesi, Indonesia, from February to April 2021. All participants who met the criteria were required to fill an informed consent sheet before being enrolled in this study. This research had obtained approval from the Research Ethics Commission of the Faculty of Medicine, Hasanuddin University (no: 112/UN4.6.4.5.31/PP36/2021).

**Patient:** a series of 32 patients with refractory GERD who met the inclusion criteria were included in analysis. Inclusion criteria were GERD patients aged 18-65 years who had undergone treatment with a proton pump inhibitor (PPI) for eight weeks with no satisfactory outcome. Exclusion criteria were pregnant women, intralaryngeal disorders such as tumors, metabolic disorders, and parasitic infections.

**Procedure:** patients who met the inclusion and not exclusion criteria underwent flexible esophagoscopy, and two samples of esophageal mucosal tissue were taken. The samples were then examined at the Anatomical Pathology Laboratory, Hasanuddin University Hospital, Makassar, South Sulawesi, Indonesia. Calculation of the number of eosinophils in peripheral blood was done by examining blood samples.

**Statistical analysis:** data analysis was done using SPSS version 23 (SPSS Inc. Chicago, IL, USA) and was statistically analyzed by Chi-Square test with a significance level of 0.05 (α = 5%).

## Results

Table 1 presents an overview of patients with eosinophilic esophagitis and non-eosinophilic esophagitis. There were two male patients (6.2%) with eosinophilic esophagitis, both of whom had a BMI between 18.5 -22.9 m/kg^2^. One patient was in the 36-45 years age group and another was in the 46-55 years aged group. The main complaint in both patients was heartburn and melena, respectively. The endoscopy result in each patient was NERD and ERD grade C, respectively. Table 2 shows the eosinophil level was < 15/HPF in 30 subjects (93.8%) and > 15/HPF in 2 subjects (6.3%).

**Table 1:** distribution of refractory GERD patients undergoing endoscopic examination

Characteristic	Non-eosinophilic esophagitis		Eosinophilic Esophagitis		%
	n	Mean ± SD	n	Mean ± SD	
		%		%	
**Sex**					
Male	14	43.8	2	6.2	50
Female	16	50	0	0	50
**Age (Years)**		42±14		45±11	
18 - 25	3	9.4	0	0	9.4
26 - 35	10	31.3	0	0	31.3
36 - 45	3	9.4	1	3.1	12.5
46 - 55	9	28.1	1	3.1	31.2
≥ 56	5	15.6	0	0	15.6
**Occupation**					
Entrepreneur	11	34.4	1	3.1	37.5
Housewife	6	18.8	0	0	18.8
Government employees	6	18.8	0	0	18.8
None	2	6.3	0	0	6.3
Others	5	15.6	1	3.1	18.7
**Subjective Complaints**					
Heartburn	20	62.5	0	0	62.5
Epigastric pain	6	18.8	1	3.1	21.9
Melena	2	18.8	1	3.1	21.9
Hematemesis	1	6.3	0	0	6.3
Dysphagia	1	15.6	0	0	15.6
**BMI (kg/m2)**		21.47±2.55		21.71±3.00	
<18.5	3	9.4	0	0	9.4
18.5-22.9	16	50.0	2	6.3	56.3
23 - 24.9	10	31.3	0	0	31.3
25 - 29.9	1	3.1	0	0	3.1
**Endoscopy**					
NERD	9	28.0	1	3.1	31.3
ERD grade A	10	31.3	0	0	31.3
ERD grade B	9	28.0	0	0	28.0
ERD grade C	2	6.3	1	3.1	9.4
**Total**	30	93,75	2	6,25	100

**Table 2:** eosinophil level of esophageal tissue

Tissue Eosinophils	n	%
< 15/HPF	30	93.8
≥15/HPF	2	6.3
Total	32	100

### Correlation between eosinophils in esophageal tissue and eosinophils in peripheral blood

Table 3 shows the distribution of peripheral blood eosinophil levels and esophageal tissue in patients with refractory GERD. No correlation between peripheral blood eosinophil level and esophageal tissue was found (p>0.466).

**Table 3:** correlation of eosinophil levels in esophageal tissue and blood in refractory GERD

Variable	Eosinophils in the blood			
	Normal		Mild eosinophilia	
	n	%	n	%
Non-eosinophilic esophagitis	20	62.5	10	31.2
Eosinophilic esophagitis	2	6.3	0	0
Total	22	68.8	10	31.2

## Discussion

The study showed that males and females had the same risk of developing refractory GERD and the 25-36 years age group was the population with the highest risk of having refractory GERD. A study by Tarigan *et al*. showed that the incidence of GERD was more in males, with the majority being over 40 years old (63.16%) [10] and was supported by another study that showed that GERD was more common in males (odds ratio [OR] 1.268) and in people older than 50 years (OR 3.179) [11]. A total of 20 subjects (62.5%) reported heartburn as the chief complaint and the majority of participants (18 subjects, 56.3%) had normal BMI (18.5 - 22.9 m/kg^2^). The most frequently obtained endoscopy results were NERD and ERD grade A (10 subjects, 31.3%, respectively). The findings by Tarigan *et al*. revealed that the most common complaint was epigastric pain (33.3%) [10]. Other studies also confirmed our result and reported that complaints were heartburn and acid regurgitation [1, 12].

The prevalence of eosinophilic esophagitis in GERD patients varies across studies. This study found that the prevalence of eosinophilic esophagitis was 6.3%. In the study of Fujiwara *et al*. in Japan, the prevalence of eosinophilic esophagitis was 0.04%. However, a study by Foroutan in Iran stated that the prevalence of eosinophilic esophagitis in patients with refractory GERD was 8.8%. Garcia *et al*. found a 4% prevalence of eosinophilic esophagitis, which was similar to our data [13, 14]. This study revealed that all patients with eosinophilic esophagitis were male, with one person aged between 36-45 years and another aged between 46-55 years. Research conducted by Garcia in Mexico found no difference in the incidence of eosinophilic esophagitis in males and females with the age group of under 45 years (83.3%). However, a Canadian study stated that the incidence of eosinophilic esophagitis was higher in men in the second decade of life (mean age 31.8 ± 17.7 years). Findings by Foroutan *et al*. in Iran showed the prevalence of eosinophilic esophagitis was higher in the female with an age range of 23-44 years (mean of 41.29±3.33 years) which was supported by Anis *et al*. in Pakistan who found that eosinophilic esophagitis was more common in women than men (5: 3) with an age range of 41-63 years [14-16]. These results suggest that the role of gender in eosinophilic esophagitis is still unclear.

The main complaint of the two cases of eosinophilic esophagitis in this study was heartburn and melena, respectively. Garcia *et al*. revealed that 100% patients with eosinophilic esophagitis had dysphagia while heartburn was felt in 3 patients (50%). Similarly, other studies by Mulder *et al*. in Canada and Anis in Pakistan showed that the chief complaint was dysphagia (74%) [14-16]. A research conducted by Fujiwara *et al*. in Japan in 2012 found 7 cases of eosinophilic esophagitis (9.9%) of 13,634 subjects who underwent upper GI tract endoscopy which was predominated by men with the main complaints of dysphagia and heartburn [13]. According to the data of this study, no increase in peripheral blood eosinophilia was found in all subjects, including those with eosinophilic esophagitis. Based on research conducted by Mulder *et al*. in Canada, there was an increase in the number of eosinophils with a mean of 0.35 ± 0.29/L [17]. At the same time, Yasuhiko *et al*. in Japan in 2017 stated that the increase in blood eosinophil levels in patients with eosinophilic esophagitis was only around 10 - 30% [18]. According to the study of Kinoshita *et al*. in Japan, the increased level of eosinophils in the peripheral blood was observed in 34.6% of eosinophilic esophagitis patients (26 patients out of 170 eosinophilic gastroenteritis patients) and concluded that the association between increased eosinophil level in the blood and eosinophilic esophagitis was still unclear [19]. It has to be noted that the involvement of other gastrointestinal organs and symptoms of atopy can further trigger an increase in the number of eosinophils in the blood [18, 19].

The correlation between eosinophils in peripheral blood and the esophagus has also been studied by Konikoff *et al*. in Ohio, United States. According to Konikoff, there is a significant correlation between an increase of eosinophils in the blood and esophageal tissue. However, this examination can only be used as a predictor of the severity of esophagitis symptoms. It is proven that the number of eosinophils in the blood decreased after therapy, accompanied by improvement in symptoms [20]. To our knowledge, this is the first study on the clinical profile and characteristics of patients with eosinophilic esophagitis in Indonesia. Future studies should be done with larger sample size to improve our understanding on the clinical profile and pathophysiology of eosinophilic esophagitis.

## Conclusion

Symptoms complained of by patients with eosinophilic esophagitis are similar to with refractory GERD. Therefore, it is imperative to take thorough history and investigations. The increase in eosinophils in the peripheral blood does not necessarily occur in all patients with eosinophilic esophagitis.

### What is known about this topic

- Refractory GERD is a typical GERD that does not respond to twice daily administration of proton pump inhibitor (PPI) for 4-8 weeks;
- The diagnosis of eosinophilic esophagitis is made based on the clinical symptoms and the presence of > 15 eosinophils per high power field (HPF) in the esophageal mucosa from esophageal biopsy, especially in the mid or proximal esophagus;
- The clinical manifestation of eosinophilic esophagitis is indistinguishable from refractory GERD.

### What this study adds

- The prevalence of eosinophilic esophagitis in patients in presenting with refractory GERD is 6.3%;
- No association between blood eosinophil and esophageal tissue eosinophil level was found in patients with eosinophilic esophagitis;
- We recommend conducting esophageal biopsy in patients with refractory GERD.

## References

[1] Clarrett DM, Hachem C (2018). Gastroesophageal Reflux Disease (GERD). Mo Med.

[2] Al-Ani M, Winchester DE (2015). Prevalence and Overlap of Noncardiac Conditions in the Evaluation of Low-risk Acute Chest Pain Patients. Crit Pathw Cardiol.

[3] Naik RD, Meyers MH, Vaezi MF (2020). Treatment of refractory gastroesophageal reflux disease. Gastroenterol Hepatol (NY).

[4] Mermelstein J, Mermelstein AC, Chait MM (2018). Proton pump inhibitor-refractory gastroesophageal reflux disease: challenges and solutions. Clin Exp Gastroenterol.

[5] Fock KM, Talley NJ, Fass R, Goh KL, Katelaris P, Hunt R (2008). Asia-Pacific consensus on the management of gastroesophageal reflux disease: update. J Gastroenterol Hepatol.

[6] Richter JE (2014). Current Diagnosis and Management of Suspected Reflux Symptoms Refractory to Proton Pump Inhibitor Therapy. Gastroenterol Hepatol (N Y).

[7] Arora AS, Yamazaki K (2004). Eosinophilic esophagitis: asthma of the esophagus?. Clin Gastroenterol Hepatol.

[8] Gomez Torrijos E, Gonzalez-Mendiola R, Alvarado M, Avila R, Prieto-Garcia A, Valbuena T (2018). Eosinophilic Esophagitis: Review and Update. Frontiers in medicine.

[9] Spergel JM (2007). Eosinophilic esophagitis in adults and children: evidence for a food allergy component in many patients. Curr Opin Allergy Clin Immunol.

[10] Tarigan R, Pratomo B (2019). Gastroesophageal Reflux Risk Factor Analysis at Saiful Anwar Hospital in Malang. Jurnal Penyakit Dalam Indonesia.

[11] Syam AF, Sobur CS, Hapsari F, Abdullah M, Makmun D (2017). Prevalence and Risk Factors of GERD in Indonesian Population-An Internet-Based Study. Advanced Science Letters.

[12] Richter JE, Rubenstein JH (2018). Presentation and Epidemiology of Gastroesophageal Reflux Disease. Gastroenterology.

[13] Fujiwara Y, Sugawa T, Tanaka F, Tatsuwaki H, Okuyama M, Hayakawa T (2012). A multicenter study on the prevalence of eosinophilic esophagitis and PPI-responsive esophageal eosinophilic infiltration. Intern Med.

[14] Foroutan M, Norouzi A, Molaei M, Mirbagheri SA, Irvani S, Sadeghi A (2010). Eosinophilic esophagitis in patients with refractory gastroesophageal reflux disease. Dig Dis Sci.

[15] Anis K, Chandnani A, Ahmed MU, Shaukat F (2019). Retrospective analysis of eosinophilic esophagitis in patients with refractory gastroesophageal reflux disease. Cureus.

[16] García-Compeán D, González JAG, García CAM, Gutiérrez JPF, Quintana OB, Rodríguez GG (2011). Prevalence of eosinophilic esophagitis in patients with refractory gastroesophageal reflux disease symptoms: a prospective study. Dig Liver Dis.

[17] Mulder DJ, Hurlbut DJ, Noble AJ, Justinich CJ (2013). Clinical features distinguish eosinophilic and reflux-induced esophagitis. J Pediatr Gastroenterol Nutr.

[18] Abe Y, Sasaki Y, Yagi M, Yaoita T, Nishise S, Ueno Y (2017). Diagnosis and treatment of eosinophilic esophagitis in clinical practice. Clin J Gastroenterol.

[19] Kinoshita Y, Furuta K, Ishimaura N, Ishihara S, Sato S, Maruyama R (2013). Clinical characteristics of Japanese patients with eosinophilic esophagitis and eosinophilic gastroenteritis. J Gastroenterol.

[20] Konikoff MR, Blanchard C, Kirby C, Buckmeier BK, Cohen MB, Heubi JE (2006). Potential of blood eosinophils, eosinophil-derived neurotoxin, and eotaxin-3 as biomarkers of eosinophilic esophagitis. Clin Gastroenterol Hepatol.

